# Inhaled corticosteroids in COPD: Personalising the therapeutic choice

**DOI:** 10.7196/AJTCCM.2018.v24i1.184

**Published:** 2018-04-03

**Authors:** J A Shaw, E M Irusen

**Affiliations:** Division of Pulmonology, Department of Medicine, Tygerberg Academic Hospital and Stellenbosch University, Cape Town, South Africa

**Keywords:** COPD, inhaled corticosteroids

## Abstract

There has been a recent surge in interest in the role of inhaled corticosteroids (ICS) in the treatment of COPD, especially regarding patients with high eosinophil counts. Evidence has shown that despite the increase in localised adverse effects and a small increase in non-fatal pneumonia events with ICS use, ICS still have an important role to play in reducing exacerbation rates and addressing the inflammation that is at the heart of the pathogenesis of COPD. Current international guidelines recommend the use of ICS only in patients with severe disease. This review examines the potential role of ICS in all COPD patients.

## Background

The use of inhaled corticosteroids (ICS) in chronic obstructive pulmonary disease (COPD) remains a topic of contention among doctors and data on the subject are often contradictory.^[1]^ Recently, there has been a trend toward down-playing ICS use in COPD treatment regimens in all but the most severe group of patients.^[2,3]^ The current Global Initiative for Chronic Obstructive Lung Disease (GOLD) strategy document^[4]^ suggests that ICS should only be used in GOLD C and D patients (i.e. those with two or more exacerbations or one exacerbation leading to hospital admission). There are two primary reasons given against ICS use in other categories of COPD. Firstly, the GOLD strategy cites *in vitro* evidence that the inflammation present in COPD is inherently corticosteroid resistant. The second concern raised is the highly topical increased risk of nonfatal pneumonia in patients with severe COPD who use ICS.

Here, we review the evidence for the abovementioned assertions by examining data on the efficacy of ICS in COPD, the pharmacological actions of ICS with relation to the pathogenesis of COPD, as well as examining the strength of the evidence for an increased risk of pneumonia in this population. We conclude with recommendations on the use of ICS.

## Known clinical effects of ICS in COPD

In patients with COPD, exacerbations are associated with an increased risk of mortality, poorer quality of life, and accelerated long-term decline in lung function.^[5,6]^ These effects are greater in those who experience such events more frequently.^[7,8]^ There is good evidence to show that long-term use of ICS reduces the rate of exacerbations in patients with both moderate and severe airflow limitation.^[9,10]^

ICS use has also been shown to affect patients’ quality of life (QOL) and symptoms. In a meta-analysis of ICS use for stable COPD, the rate of decline in QOL as measured by the St George’s Respiratory Questionnaire (SGRQ) was reduced, and there was a small, but statistically significant, improvement in patients’ QOL.^[9]^ In this same meta-analysis it was noted that some studies also showed a reduction in rescue bronchodilator use.^[9]^

The Withdrawal of Inhaled Steroids during Optimized Bronchodilator Management (WISDOM) study by Magnusson *et al*. ^[11]^ suggested a slower rate of decline in the forced expiratory volume in one second (FEV_1_ ) in patients who received ICS therapy; however, long-term use of ICS has not consistently been shown to reduce the rate of decline in FEV_1_ , or to have any significant effect on mortality in COPD patients.^[9]^ These observations were most recently corroborated in the Study to Understand Mortality and MorbidITy in COPD (SUMMIT).^[12]^

While ICS are currently recommended for patients with an FEV_1_ value <60% of predicted and a history of exacerbations, the SUMMIT sub-study suggests that ICS may also have a role in other patient groups, as there were benefits in those with an FEV_1_ >60% of predicted and in patients with no exacerbation history.^[10]^

## Combination therapy and evidence for synergistic effects

A meta-analysis of treatment options for patients with severe COPD who remained uncontrolled on short-acting muscarinic-antagonists (SAMA) and short-acting beta-agonists (SABA) alone, found that a combination of an ICS and long-acting beta-agonist (LABA) was the highest-ranked intervention for improving QOL compared with placebo at 6 and 12 months.^[13]^ Long-acting muscarinic-antagonist (LAMA) and LABA therapy were independently ranked second and third, and ICS alone was ranked fourth at 6 months. Martinez *et al*. ^[10]^ recently demonstrated that the combination of ICS/LABA reduced exacerbation rates to a greater degree than either component alone. It has been proposed that the mechanism of this synergistic effect is the LABA enhancing glucocorticoid receptor nuclear translocation and efficacy. This was demonstrated in a study of induced-sputum macrophages: the combination of salmeterol and 100 µg fluticasone propionate (FP) significantly increased nuclear glucocorticoid receptor levels equivalent to that of 500 µg FP, enhanced ICS-induced mitogen-activated protein kinase phosphatase-1 (MAPK1) mRNA copies and doubled glucocorticoid response element-luciferase reporter gene activity.^[14]^ There is also evidence that the budesonide/ formoterol combination enhanced the expression of pro-surfactant protein-B in the lungs of COPD patients – a population in which surfactant expression is decreased and which has also been associated with poor health outcomes.^[15]^

## Triple therapy

Recently, data have emerged regarding the so-called ‘triple therapy’, which includes a combination of ICS/LAMA/LABA treatment.

Clinical trials have previously tested the effectiveness of triple therapy delivered by two separate devices, compared to LAMA monotherapy, LAMA/LABA combination therapy using separate inhalers, and combined ICS/LABA treatment. These studies showed a short-term superiority of triple therapy in terms of lung function and patient-reported outcomes when compared with LAMA monotherapy or ICS/LABA treatment.^[16]^

One study that compared triple therapy with LAMA/LABA (tiotropium and salmeterol) combination therapy (the latter group having had the ICS (FP) sequentially decreased and then completely withdrawn from the initial triple regimen) noted no significant difference in the exacerbation rate. However, they did observe a significant decrease in FEV_1_ in the group in which ICS was withdrawn, as well as a worsening of dyspnoea scores and health status outcomes.^[11]^ It is important to note that this was a non-inferiority study and thus equivalence or superiority cannot be presumed.

Two large randomised trials have compared the ICS/LAMA/ LABA combination in a single inhaler device with ICS/LABA, with similar results: in patients with severe COPD, triple therapy was found to be superior to ICS/LABA combination in improvements in FEV_1_ , reduction in exacerbation rate, as well as health-related QOL scores. ^[16,17]^ In TRILOGY, there was a 23% reduction in exacerbations with extra-fine beclomethasone dipropionate, formoterol furoate and glycopyrronium bromide (BDP/FF/GB) compared with BDP/FF.^[16]^ In FULFIL (Lung FUnction and quality of LiFe assessment in COPD with closed trIpLe therapy), the addition of umeclidinium to FF/VI resulted in a net FEV_1_ gain of 179 mL compared with BDP/ FF at 1 year, with a higher percentage of subjects who were SGRQ responders in the former and a mean SGRQ change of –4.6 units compared with -1.9 U with BDP/FF.^[17]^ Such a clinically significant difference in the SGRQ (–4U is the clinically significant threshold that patients can perceive) has seldom been documented in COPD trials.

Currently, there are no good-quality prospective data comparing triple therapy with the LABA/LAMA combination.^[18]^

## Withdrawal of ICS

A recent meta-analysis on the effects of withdrawal of ICS showed that, while ICS withdrawal did not significantly increase the overall rate of COPD exacerbations, a clinically important increased risk of severe exacerbation was detected. ICS withdrawal significantly impaired both lung function and QOL. The time to the first exacerbation was also significantly shorter in the patients who discontinued ICS.^[19]^

## Corticosteroids, inflammation and COPD

It is well known that inflammation of the airways is present even in the early stages of COPD.^[20]^ The dominant inflammatory cells are neutrophils; however, increased numbers of macrophages and CD8^+^ T lymphocytes are also present, all of which interact to produce chemokines, cytokines, proteases and reactive oxygen species that cause tissue damage and stimulate further inflammation.^[21,22]^ The presence of inflammatory biomarkers in the sputum has been associated with disease progression and an increased risk of exacerbations,^[23]^ while suppression of airway inflammation has been shown to improve lung function^[24]^ and reduce exacerbation rates by up to 30%.^[25]^

Corticosteroids suppress the multiple inflammatory genes that are activated in chronic inflammatory diseases, such as COPD. This is achieved mainly by reversing histone acetylation of activated inflammatory genes through binding of liganded glucocorticoid receptors to coactivators, and recruitment of histone deacetylase-2 (HDAC2) to the activated transcription complex.^[26]^

It has been suggested that the inflammation specific to COPD is resistant to corticosteroid effects, possibly through reduced HDAC2 expression.^[27]^ However, it has been demonstrated that the action of budesonide in suppressing airway inflammation is independent of the HDAC2 pathway.^[28]^ Other postulated mechanisms of ICS resistance in COPD include activation of mitogen-activated protein (MAP) kinase pathways by certain cytokines, excessive activation of the transcription factor activator protein 1, raised macrophage migration inhibitory factor, and increased P-glycoprotein-mediated drug efflux.^[29]^ However, a meta-analysis of studies examining inflammatory biomarkers in sputum, bronchoalveolar lavage fluid and biopsy specimens concluded that ICS were effective in reducing CD4^+^ and CD8^+^ T cell counts, as well as neutrophil and lymphocyte counts. It was noted that macrophage counts were increased in the presence of ICS. The authors hypothesised that these important immunomodulatory effects could be the reason for the efficacy of ICS in reducing exacerbations, as well as the mechanism underlying the apparent increase in pneumonia.^[29]^ A subsequent study concluded that even in the presence of smoking, long-term ICS treatment may lead to anti-inflammatory effects in the lung as ICS reduced bronchial mast cells, CD3^+^, CD4^+^ and CD8^+^ cells, as well as sputum neutrophils and lymphocytes.^[30]^ In addition, a recent report has demonstrated that ICS discontinuation in patients on long-term ICS with moderate-to-severe COPD resulted in increased airway inflammation, as reflected by increased numbers of bronchial CD3^+^, CD4^+^, and CD8^+^ T cells and mast cells, as well as increased sputum total cell count, macrophages, neutrophils and lymphocytes.^[31]^

Another study identified an ICS-insensitive macrophage phenotype in COPD. These macrophages showed significantly lower expression of all receptors, and were associated with higher levels of release of active matrix metalloproteinase 9 compared with macrophages of non-smokers and smokers without COPD.^[32]^ A COPD phenotype that is more likely to respond to ICS has not yet been identified, as response is not predicted by oral steroid response, bronchodilator reversibility or bronchial hyper-responsiveness.^[32]^ However, there is evidence that long-term benefits of ICS on lung function decline in patients with moderate-to-severe COPD are most pronounced in patients with fewer pack years smoking history, less severe emphysema (limited hyperinflation and preserved diffusion) and lower sputum inflammatory cell counts.^[33]^

## Eosinophilic inflammation in COPD

A *post hoc* analysis of two large multinational studies comparing treatment with ICS/LABA (fluticasone fuorate/vilanterol[VI]) to VI monotherapy, found that patients with a blood eosinophil count ≥2.4%, responded better to the combination, with a generally linear relationship of further exacerbation reduction with higher eosinophil counts. The inference from their analysis was that, in general, low eosinophil counts coupled with high levels of smoking could predict a poorer response to ICS, with no significant reduction in exacerbation rates.^[34]^ The linear association of blood eosinophils with exacerbation reduction by ICS was also noted in a further analysis of the WISDOM study using tiotropium, salmeterol and FP.^[35]^ In a *post hoc* analysis of the INSPIRE (Investigating New Standards for Prophylaxis in Reduction of Exacerbations) study using an eosinophil cut-off of 2%, FP/salmeterol was associated with a 25% relative risk reduction of exacerbations compared with tiotropium alone.^[36]^ This, and other evidence regarding the anti-inflammatory effects of ICS in COPD, is captured in Table 1^[40–45]^.

**Table:** Key studies identifying the anti-inflammatory effects of ICS in COPD

**Study**	**Findings**
Thompson, 1992^[37]^	Reduction in bronchoalveolar lavage fluid cellularity, lactoferrin, lyzozyme and albumin levels (markers of inflammation).
Saetta, 1997;^[38]^ Saetta, 1998^[39]^	The key inflammatory cells mediating inflammation in COPD were CD68^+^ macrophages, neutrophils and CD8^+^ cytotoxic lymphocytes.
Bhowmik, 2000^[23]^	Inflammatory biomarkers in the sputum were associated with disease progression and an increased risk of exacerbation.
Cosio, 2002^[22]^	Neutrophils were the dominant airway inflammatory cells in COPD.
Barnes, 2003^[21]^	Macrophages and CD8^+^ T lymphocytes were also increased.
	Above cells interacted to cause tissue damage and further inflammation.
Hattotuwa, 2002^[40]^	Reduction in CD8:CD4 ratio.
	No reduction in CD8^+^, CD68^+^ cells or neutrophils observed, suggested that ICS worked on specific aspects of airway inflammation.
Sugiura, 2003^[24]^	Suppression of airway inflammation improved lung function.
Sin, 2003^[25]^	Suppression of airway inflammation reduced exacerbation rates.
Hogg, 2004^[22]^	Inflammation of the airways was present even in the early stages of COPD.
Ozol, 2005^[41]^	Reduction in interleukin (IL)-8 levels in bronchoalveolar lavage fluid mean percentage of neutrophils.
Gan, 2005^[42]^ (meta-analysis)	Reduction in sputum total cell, neutrophil and lymphocyte counts when given in adequate dose and duration.
Barnes, 2006^[43]^	Reduction in CD8^+^, CD45^+^ and CD4^+^ cells, but no change in CD68^+^ cells seen.
Bathoorn, 2008^[44]^	Reduction in sputum eosinophilia.
Lapperre, 2009^[45]^	Reduction in counts of mucosal CD3^+^, CD4^+^, CD8^+^ and mast cells, with effects maintained after 30 months.
Jen, 2012^[29]^ (meta-analysis)	Reduction in CD4^+^ and CD8^+^ T cell counts, as well as neutrophil and lymphocyte counts in bronchoalveolar lavage fluid and biopsy specimens.
Wang, 2013^[28]^	Action of budesonide on airway inflammation was independent of the HDAC2 pathway.
Hoonhorst, 2014^[30]^	Reduction in bronchial mast cells, CD3^+^, CD4^+^ and CD8^+^ cells, as well as sputum neutrophils and lymphocytes.
Chana, 2014^[32]^	An ICS insensitive macrophage phenotype identified (lower expression of all receptors, higher levels of release of active matrix metalloproteinase 9).
Snoeck-Stroband, 2015^[33]^	Long-term benefits on lung function decline in patients with moderate-to-severe COPD were most pronounced in patients with fewer pack years smoking history, less severe emphysema and lower sputum inflammatory cell counts.
Hinds, 2016^[34]^	Patients with blood eosinophil counts ≥2.4% responded better to ICS/LABA.
	A linear relationship of further exacerbation reduction with higher eosinophil counts was found.
	Low eosinophil counts coupled with high levels of smoking could predict a poorer response to ICS, with no significant reduction in exacerbation rates.
Watz, 2016^[35]^	Higher blood eosinophils associated with reduction in exacerbation rate in a linear relationship.
Pavord, 2016^[36]^	Blood eosinophils of >2% associated with a 25% relative risk reduction of exacerbations with ICS use.
Kunz, 2017^[31]^	Discontinuation resulted in increased numbers of bronchial CD3^+^, CD4^+^, and CD8^+^ T cells and mast cells, as well as increased sputum total cell count, macrophages, neutrophils and lymphocytes.

ICS = inhaled corticosteroids

COPD = chronic obstructive pulmonary disease

LABA = long-acting beta agonist

## The risk of pneumonia

There is no doubt that the use of ICS is associated with an increased risk of localised adverse effects: oropharyngeal candidiasis, dysphonia and hoarseness, as well as an increased risk of cataracts.^[32,46]^ Additionally, ICS increase the risk of non-fatal serious adverse pneumonia events, without conferring any difference in overall mortality rate.^[47]^ This effect was first unexpectedly identified in the large prospective TORCH (TOwards a Revolution in COPD Health) study,^[48]^ and has subsequently been shown in numerous large randomised trials (Table 2)^[49–63]^ and smaller studies. Recent studies have reported a stronger association with pneumonia at higher doses of ICS, suggesting a dose-response relationship, and age older than 65 has been identified as an additional factor which increases risk.^[46]^ However, it is worth noting that the incidence of pneumonia in all the above studies is low (<6% overall and usually between 0% and 2% more than placebo/LABA). The latter is a reminder that COPD itself predisposes patients to pneumonia through an altered microbiome and the toxic effects of cigarette smoking, as well as the fact that the disease occurs in older individuals who may have used oral corticosteroids, which leads to further suppression of the immune response.

**Table 2:** The effect of ICS use in COPD on pneumonia risk in important clinical trials

**Study**	**Population**	**Intervention (*N*)**	**Effect on pneumonia risk**
**TORCH** Calverly, 2007^[48]^	Moderate to severe obstruction	Placebo (851) LABA (960) ICS (947) ICS/LABA (1 011)	Risk of pneumonia over 3 years 18.3% and 19.6% in ICS/LABA and ICS groups v. 12.3% and 13.3% in placebo and LABA groups (*p*<0.001). No increase in risk of death from pneumonia in ICS groups.
**SHINE** Tashkin, 2008^[49]^	Severe obstruction with exacerbations	Placebo (300) ICS (275) LABA (284) ICS/LABA (845)	No increase in risk compared with placebo.
**INSPIRE** Wedzicha, 2008^[50]^ Calverly, 2011 reanalysis^[51]^	Severe obstruction with exacerbations	ICS/LABA (658) LAMA (665)	Hazard ratio of 1.94 for having pneumonia in ICS group (*p*=0.008); unchanged when analysis restricted only to patients with CXR. Higher risk in those with baseline severe dyspnoea and baseline raised CRP.
Dransfield *et al*., 2013^[52]^	COPD with exacerbations	LABA (818)	Incidence of non-fatal pneumonia increased in ICS group.
		ICS/LABA (3 dose variations 820/806/811)	High-dose ICS discontinued.
**ILLUMINATE, LANTERN** Pooled analysis Vogelmeier, 2013^[53]^ Zhong, 2015^[54]^ Vogelmeier, 2016^[55]^	Moderate to severe obstruction	LABA/LAMA (259 and 372) ICS/LABA (264 and 369)	Incidence of pneumonia 0.5% in LABA/LAMA group and 2.2% in the ICS/ LABA group (*p*=0.0074). Higher rate in more severe COPD.
**FORWARD** Wedzicha, 2014^[56]^	Severe obstruction with exacerbations	ICS/LABA (595) LABA (591)	Incidence of pneumonia 1.8% in LABA group and 3.8% in ICS/LABA group.
**INSTEAD** Rossi, 2014^[57]^	Moderate obstruction	ICS/LABA (250) LABA (246)	Incidence of pneumonia 0% in LABA group and 0.7% in ICS group, not statistically significant.
**WISDOM** Magnussen, 2014^[11]^	Severe obstruction with exacerbations	ICS/LABA/LAMA (1 243) LABA/LAMA (1 242)	Hazard ratio for time to first exacerbation = 1.06. Incidence of pneumonia increased from 5.5% to 5.8% in ICS group, not statistically significant.
**SALFORD** Vestbo, 2016^[58]^	COPD with excerbations	ICS/LABA (1 396) Usual care (1 403)	No excess pneumonia risk.
**TRILOGY** Singh, 2016^[15]^	Severe obstruction with exacerbations	ICS/LABA/LABA (687) ICS/LABA (681)	Incidence of pneumonia in both groups = 3%
**FLAME** Wedzicha, 2016^[59]^	Moderate to severe obstruction with exacerbations	LABA/LAMA (1 675) ICS/LABA (1 679)	Incidence of pneumonia 3.2% in LABA/LAMA group and 4.8% in ICS/ LABA group (*p*=0.02).
**IMPACT** Wang, 2016^[60]^	COPD with ICS use	Pneumonia cases (19 838) Pneumonia controls (74 849)	Odds ratio for pneumonia = 1.25. Odds increased with increasing ICS dose.
**UPLIFT** Tashkin, 2008^[61]^ Morjaria, 2017 reanalysis^[62]^	Moderate to severe obstruction	No ICS (2 292) Fluticasone roprionate (1 981) Other ICS (1 719)	Incidence of pneumonia 5.6% in no-ICS group and 6.8% with ICS use, and was higher with fluticasone proprionate than ‘other ICS’ (*p*=0.012).
**OUTPUL** Di Martino, 2014^[63]^ Cascini, 2017 reanalysis^[46]^	Moderate to severe COPD	Pneumonia cases (3 141) Pneumonia controls (12 564)	Relative risk of pneumonia = 2.29 with current ICS use. Relative risk of pneumonia = 1.23 with past ICS use. Risk rates increased with increasing dose of ICS and with increasing age. ICS users had a numerically lower risk of death.
**FULFIL** Lipson, 2017^[17]^	Moderate to severe obstruction with exacerbations	ICS/LABA/LAMA (911) ICS/LABA (899)	At 52 weeks the groups had a risk of pneumonia of 1.9% and 1.8%.

ICS = inhaled corticosteroids

COPD = chronic obstructive pulmonary disease

LABA = long-acting beta agonist

LAMA = long-acting muscarinic antagonist

CXR = chest radiograph

CRP = C-reactive protein

The prevailing hypothesis for the mechanism of the propensity to pneumonia with ICS is local airway immunosuppression and a diminished innate immune response to pathogens. Paradoxically, this diminished inflammatory effect is also hypothesised to be the mechanism for the lack of severity of these pneumonia events and thus the low fatality rate.^[64,65]^ The overall quality of the studies dedicated to examining the treatment of COPD is high. There are a number of well-conducted randomised controlled trials with large patient numbers available for analysis. However, there have been a few criticisms of the studies from which the association between ICS use and pneumonia is drawn.

The first criticism is a methodological one regarding differences in trial design, patient population and the type of interventions, all of which combine to increase differences in the reported rate of pneumonia events between studies.^[66]^ Dransfield *et al*. ^[52]^ reported a twofold increase in the pneumonia events in the combination ICS/LABA (FF/VI) arm compared with the LABA (VI) monotherapy arm in patients with at least one exacerbation in the previous year and severe airflow obstruction. They recruited >800 patients in each arm of the study, which in another context would be considered a large study. However, the recent SUMMIT trial included >4 000 patients in each arm (FF/VI v. the monotherapy components and placebo), with only moderate airflow obstruction, and reported that the difference in pneumonia events between the combination and the placebo arm was not statistically significant.^[10]^ These two studies highlight the difficulties with interpretation of the existing body of evidence: two large studies reporting on the same outcome, about the same drugs, but with different patient populations, vastly different numbers and opposing conclusions.

The second criticism is a ls o methodological in nature, and refers to the case definition (or lack thereof) used in the reporting of pneumonia events in these trials. The analysis of pneumonia events with ICS therapy in COPD is complicated by the overlap in clinical features of COPD exacerbations and pneumonia, and by the fact that pneumonia remains a relatively uncommon event when compared with acute exacerbations. While the majority of these trials were randomised controlled trials and had the highest quality evidence, they did not adhere to any formal definitions of pneumonia events. Rather, they relied on either the investigator’s retrospective assessment of a reported adverse respiratory event or database reporting systems. Chest radiographs were also not routinely performed or assessed at the time of the reported events.^[66]^

## Conclusion

The recent suggestion that LAMA/ LABA should be the baseline treatment of all patients from GOLD B-D raises the following two concerns:

- if inflammation is at the core of the pathogenesis of COPD, then this important component is not being addressed with bronchodilators alone; and
- does combination therapy with LAMA/LABA represent the ceiling effect, that is, can no further therapeutic gain be achieved?

The available evidence shows that the use of ICS in patients with COPD does confer additional clinically significant beneficial effects, in particular the reduction of exacerbations. While the inflammation that occurs in COPD may be partially corticosteroid resistant, there is good evidence that the use of ICS reduces airway inflammation in a meaningful way. Furthermore, the data suggesting that certain COPD phenotypes will derive benefit from ICS, particularly patients with blood eosinophilia ≥2% or 150 cells/µL, is accumulating rapidly.^[34]^ The search for a more reliable biomarker of the phenotype of ICS responsiveness is still underway.

An increase in the risk of non-fatal pneumonia events has been documented; however, important methodological differences should be considered when interpreting these trials. It is worth noting that COPD patients in these studies have a baseline risk of pneumonia of up to 5.6% and the increase in risk is <2% (Table 2). It has been pointed out by other authors^[67]^ that, in considering ICS use, the riskbenefit should be carefully weighed. In one report, the exacerbation reduction with ICS use was in the order of 190 events, compared with the minor increase of ~30 pneumonia events.^[67]^ These and other data prompted the European Medicines Agency’s Pharmacovigilance Risk Assessment Committee (PRAC) report to state that, while increased risk of pneumonia remains a common side-effect for all inhaled corticosteroids, the benefits of ICS continue to outweigh the risk.^[68]^

Finally, given the promising nature of the emerging literature on triple therapy, there is the possibility this may become the treatment of choice in GOLD D patients, and may have a role in other categories of severity as well.

More research is needed to identify the true ICS-responsive phenotype, as well as to assess the effects of triple therapy in early stage COPD on lung function decline, as well as exacerbation rates. Careful attention will need to be paid to the rates of pneumonia and other adverse events in these patient groups so that an accurate riskbenefit assessment can be made, on an individual patient basis.
